# Clinical, Hormonal, and Neuroradiological Characteristics and Therapeutic Outcomes of Prolactinomas in Children and Adolescents at a Single Center

**DOI:** 10.3389/fendo.2020.00527

**Published:** 2020-08-04

**Authors:** Aram Yang, Sung Yoon Cho, Hyojung Park, Min Sun Kim, Doo-Sik Kong, Hyung-Jin Shin, Dong-Kyu Jin

**Affiliations:** ^1^Department of Pediatrics, Kangbuk Samsung Hospital, Sungkyunkwan University School of Medicine, Seoul, South Korea; ^2^Department of Pediatrics, Samsung Medical Center, Sungkyunkwan University School of Medicine, Seoul, South Korea; ^3^Department of Neurosurgery, Samsung Medical Center, Sungkyunkwan University School of Medicine, Seoul, South Korea

**Keywords:** prolactinoma, pituitary adenoma, combined pituitary hormone deficiency, dopamine agonists, transphenoidal approach

## Abstract

**Background/Purpose:** A prolactinoma is the most common pituitary adenoma, but it is relatively rare in childhood and adolescence. There is only limited research about the clinical spectrum, treatment, and outcomes of prolactinomas in childhood and adolescence. In this single-center cohort study, we assessed the clinical, hormonal, and neuroradiological characteristics and therapeutic outcomes of children and adolescents with prolactinomas.

**Methods:** This retrospective cohort study included 25 patients with prolactinomas diagnosed before 19 years of age, who presented at Samsung Medical Center during a 15-year period (March 2005 to August 2019).

**Results:** The median age at diagnosis was 16.9 (range 10.1–18.5) years, and 80% of the patients were female. The common clinical manifestations at diagnosis were galactorrhea (10/20, 50%) and amenorrhea (9/20, 45%) among females and visual field defects (3/5, 60%) and headaches (2/5, 40%) among males. In our cohort, macroadenomas accounted for 56% of cases, and the rate of overall responsiveness to dopamine agonists (DAs) was 56% (10/18). Male gender, the prolactin (PRL) level at diagnosis, and the presence of panhypopituitarism were positively correlated with maximum tumor diameter (*r* = 0.443, *P* = 0.026; *r* = 0.710, *P* < 0.001; and *r* = 0.623, *P* = 0.001, respectively). After the trans-sphenoidal approach (TSA), 53% (8/15) of patients showed normalization of the PRL level. Three patients, who underwent gamma knife surgery (GKS) owing to either resistance or intolerance to DAs or recurrence after the TSA, achieved a normal PRL level accompanied with marked tumor reduction and symptom remission.

**Conclusions:** A macroprolactinoma is more prevalent than a microprolactinoma in children and adolescents than in adults. Male gender, increased PRL levels, and the presence of panhypopituitarism at diagnosis are closely related to macroprolactinomas in children and adolescents.

## Introduction

A prolactinoma is the most common pituitary adenoma, which is characterized by lactotroph cells secreting prolactin (PRL) and monoclonal expansion of single cells in the pituitary (1). It usually occurs sporadically; however, it is dominantly inherited with germline mutations in the *AIP* or *MEN1* gene in ~5% of cases (2, 3). It constitutes around 50% of all pituitary adenomas in adults and occurs most frequently in women aged 20–50 years (4–6). However, a pediatric prolactinoma is rare, with an incidence of 0.1 per 1,000,000 population, and it accounts for <2% of all intracranial tumors (7, 8). Clinical characteristics of prolactinoma in children and adolescents may differ from those in adults, and children and adolescents are more likely to have macroprolactinomas (diameter >10 mm) compared to adults (9–11).

Clinical manifestations of a prolactinoma can result from the overproduction of prolactin and mass effects. Hyperprolactinemia inhibits the pulsatile secretion of gonadotropin-releasing hormone (GnRH) from the hypothalamus, which is required for follicle-stimulating hormone (FSH) and luteinizing hormone (LH) secretion from the pituitary gland. Lack of FSH and LH results in amenorrhea, and elevated PRL can lead to galactorrhea in pubertal girls. Macroprolactinoma, more often diagnosed in boys, may be associated with headaches, visual field defects, or other neurologic deficits that are mainly caused by mass effect (1, 12).

Dopamine agonists (DAs) are first-line treatment for children, adolescents, and adults with prolactinomas due to favorable responses. Trans-sphenoidal surgical approach (TSA) is recommended for patients who do not respond to DA, have intolerable adverse effects of DA, experience neurosurgical emergencies such as cerebrospinal fluid (CSF) leak, or experience rapid visual impairment due to pituitary apoplexy (13, 14). This approach is also considered by multiple factors, such as DA-resistant cystic prolactinoma (15), and patient preference. Recently, gamma knife surgery (GKS) emerged as a safe and effective second-line therapy for residual or recurrent pituitary adenoma. However, its clinical effects in children and adolescents remain unclear (16, 17).

Owing to disease rarity and obstacles in diagnosis and treatment, there have been only a few studies in children and adolescents with prolactinomas and accumulated data on the treatment guidelines and long-term prognosis are lacking (8, 18–20). The aims of this study were to gain more insight into and knowledge of this disease and to assess the clinical, hormonal, and neuroradiological characteristics and therapeutic outcomes for both children and adolescents with prolactinomas.

## Methods

### Patients

This study included patients with prolactinomas diagnosed before 19 years of age at Samsung Medical Center over a 15-year period (March 2005–August 2019). The diagnosis of a prolactinoma was based on typical clinical signs and symptoms, brain magnetic resonance imaging (MRI) findings, a PRL level above the normal range in at least two evaluations, and lactotroph adenoma confirmed by immunohistochemistry in patients who received TSA. Mixed adenomas were excluded in this study based on the hormonal assay (elevated serum insulin-like growth factor 1 (IGF-1) levels above the normal upper limit for gender and age) and the results of immunohistochemistry. A total of 25 patients who underwent overall pituitary hormone evaluation at the time of diagnosis, including a combined pituitary stimulation test, were included in this study.

All patients were divided into macroadenoma (>10 mm) and microadenoma (≤ 10 mm) groups according to the maximum diameter of the lesion on brain MRI. Clinical data, such as sex, age, height, weight, body mass index (BMI), menstrual history (primary or secondary amenorrhea), presence of galactorrhea (or gynecomastia in males), age at puberty, pubertal delay (Tanner stage), and presence of mass effects with headaches and/or visual disturbances, were collected retrospectively. The height standard deviation score (SDS) and BMI SDS were calculated using the 2017 growth standard for Korean children and adolescents. As this was a retrospective study, systematic genetic test could not performed. However, information on the familial history or other features related to multiple endocrine neoplasia type 1 (MEN1) was obtained from medical charts and all patients in our study were sporadic cases. This study was approved by the Institutional Review Board of Samsung Medical Center (2018-06-050).

### Endocrine Studies

All patients underwent a comprehensive endocrine evaluation at prolactinoma diagnosis. Endocrine studies included basal serum levels of prolactin, tri-iodothyronine (T3), free thyroxine (free T4), thyroid-stimulating hormone (TSH), growth hormone (GH), IGF-1, adrenocorticotropic hormone (ACTH), cortisol, LH, FSH, estradiol (females), and testosterone (males). A combined pituitary stimulation test was performed to evaluate pituitary function (cocktail test: 0.1 unit/kg regular insulin, 500 mg protirelin tartrate, and 0.1 mg gonadorelin were injected intravenously after baseline hormone sampling, with samples obtained at 30, 60, 90, and 120 min). GH deficiency was defined by a peak GH level of <3 μg/mL in an insulin stimulation test. ACTH deficiency was defined as a peak cortisol level of <180 ng/mL by the insulin tolerance test. TSH deficiency was indicated by a low basal serum free T4 level with an inappropriately normal or low TSH level (not increased by >5 mU/L) in a thyrotropin-releasing hormone stimulation test.

GnRH deficiency is difficult to differentiate from constitutional pubertal delay in childhood. We considered GnRH deficiency for patients past the mean age of puberty (females ≥13 years; males ≥14 years) if basal values for sex hormones were not in the reference range, and there was no increase of ≥2-fold at 60 min for LH and FSH [based on the response to the GnRH stimulation test and with the delay or absence of puberty (i.e., testicular volume <4 mL in males and no breast development in females)] (21). Panhypopituitarism was defined by insufficiency of more than three anterior pituitary hormones (GH, FSH, LH, ACTH, or TSH) based on the cocktail test (22). PRL levels were measured at diagnosis and then regularly at least every 3 months using an immunoradiometric assay (RIAKEY® Prolactin IRMA Tube, USA) with the DREAM GAMMA-10 analyzer (Shin Jin Medics Inc., Gyeonggi-do, Republic of Korea), with reference ranges of 1.1–13.0 and 3.5–17.9 ng/mL for males and females, respectively.

### Treatment Method for Children and Adolescents With Prolactinomas

Bromocriptine (BRC) was started at 1.25–2.5 mg/day, with doses of up to 15 mg/day per week, whereas cabergoline (CAB) was administered at an initial dose of 0.5 mg/week, followed by gradual increases of up to 3 mg/week at 2–4-week intervals until an optimal therapeutic response was achieved. After BRC and CAB were tapered to 1.25 mg/day and 0.5 mg/week, respectively, drug withdrawal was considered when the PRL level remained normal for ≥3 months and MRI showed no tumor. Endocrine remission was defined as a normal PRL level while not taking DAs for a minimum of 6 weeks. Tumor volume shrinkage was defined by a difference in tumor volume from baseline after ≥6 months of DA therapy, and it was calculated using the maximum tumor diameters (A, B, and C) in three dimensions (A × B × C/2), according to the MRI results (*n* = 22). Responsiveness to DAs was defined by a normalized PRL level and the resolution of signs and symptoms with a reduction in tumor volume of ≥50% within 6 months according to sella MRI (23, 24). Recurrence was defined as symptom relapse or tumor reappearance (confirmed by sella MRI) after remission. Resistance to DA was defined as failure to normalize serum PRL level or failure to achieve a 50% reduction in tumor size after a weekly CAB dose of at least 2 mg or 15 mg/d BRC taken for at least 6 months.

### Immunohistochemical Analysis

The standard immunohistochemical battery using antibodies to PRL, GH, ACTH, TSH, FSH, and LH, and Ki-67 assay were performed for 16 patients who underwent the TSA. Ki-67 is a protein responsible for cell proliferation throughout the cell cycle (25). The detection of Ki-67 was performed using monoclonal antibodies specific to Ki-67 (MIB-1 clone; Immunotech, Westbrook, ME, USA). To detect Ki-67, paraffin sections were heated in 10-mM citric acid (pH 6.0) for three 5-min cycles at 750 W in a microwave oven, followed by blocking in 0.5% H_2_O_2_-methanol for 10 min. The labeling index of Ki-67 was defined as the percentage of Ki-67 immunopositive cells, according to a count of 1,000 tumor cell nuclei.

### Statistical Analysis

Continuous variables are presented as mean ± standard deviation (SD) or median with interquartile range (IQR), and comparisons were performed using Student's *t*-test and the Mann-Whitney test. Spearman correlation analysis was used to assess the associations between continuous variables. A *P* < 0.05 was considered significant. Analyses were performed using SPSS Statistics software, version 23 (IBM Corp., Armonk, NY, USA).

## Results

### Characteristics of Children and Adolescents With Prolactinomas

A total of 25 patients (20 female and 5 male patients) were included in this study (Table 1). The median age at diagnosis of all patients was 16.9 (range 10.9–18.4) years. The median age at diagnosis was 12.8 years for male patients and 16.7 years for female patients. The common symptoms at diagnosis were galactorrhea (10/20, 50%) and amenorrhea (9/20, 45%) for female patients and visual field defects (3/5, 60%) and headaches (2/5, 40%) for male patients.

**Table 1:** Patient characteristics at the time of diagnosis and treatment.

	**Total subjects (*n* = 25)**	**Microadenoma (*n* = 11) (44%)**	**Macroadenoma (*n* = 14) (56%)**	*P-*value^*^
Follow-up period (yr)	3.0 (2.0, 4.1)			
Gender (female)	20/25 (80%)	11 (55%)	9 (45%)	0.134
Age at diagnosis (median, IQR) (yr)	16.9 (16.3, 18.0) (range: 10.9–18.4)	17.1 (16.7, 17.7)	16.8 (14.3, 18.1)	0.727
Height (SDS)	0.3 (−1.2, 0.7)	0.3 (−0.6, 1.7)	−1.6 (0.1, 0.7)	0.501
BMI (SDS)	0.4 (−0.2, 0.8)	0.3 (0.1, 0.4)	0.5 (−0.2, 1.3)	0.434
PRL at diagnosis (ng/mL)	207.0 (116.6, 1056.5)^†^	114.2 (85.6, 189.5)	516.0 (202.8, 3567.5)	<0.001
Maximum tumor diameter (mm)	12.0 (9.0, 21.5) (range: 4–74)	9 (7, 10)	21 (14.3, 26.3)	<0.001
Panhypopituitarism	11/25 (44%)	1/11 (9%)	10/14 (71%)	0.008
Operation (TSA)	15/25 (60%)	3/11 (27%)	12/14 (86%)	0.003
Immediate postoperative PRL levels	25.0 (2.9, 83.0)	2.7 (1.3, 2.9)	31.7 (1.2, 1,102)	0.031
Nadir PRL level (ng/mL)	9.4 (2.6, 34.5)	7.5 (0.6, 33.0)	9.7 (4.9, 43.3)	0.373
Ki-67 index	3.1 (2.0, 5.0)	3 (0.1, 5)	3.4 (1.2, 16.0)	0.536
Responsiveness to DAs	10/18 (56%)	5/7 (71%)	5/11 (45%)	0.436
At age of first use with CAB (yr)	17.5 (16.3, 19.6)	18.4 (17.2, 19.6)	16.6 (13.5, 19.5)	0.228
CAB peak (mg/wk)	1.5 (1.0, 2.0)	1.0 (1.0, 1.9)	2.0 (1.1, 2.4)	0.130
CAB duration (yr)	1.1 (0.1, 2.2)	0.3 (0.1, 1.1)	1.8 (0.4, 2.7)	0.081
BRC peak (mg/day)	30 (3.4, 105.0)	7.5	52.5 (3.1, 140)	1.000
BRC duration (yr)	1.9 (1.1, 7.6)	0.9	2.1 (1.4, 8.2)	0.333

*Data are expressed as median (IQR) or mean ± sd*.

*BMI, body mass index; IQR, interquartile range; SD, standard deviation; PRL, prolactin; TSA, transphenoidal approach; DAs, dopamine agonists; CAB, cabergoline; BRC, bromocriptine*.

* *Significant association was classified as P < 0.05*.

† *Reference range of serum prolactin: 72–10,000 ng/mL*.

Among the 25 patients, 14 (56%) had macroadenomas and 11 (44%) had microadenomas. Nine female patients showed macroprolactinomas (9/20, 45%), and all male patients had macroprolactinomas (5/5, 100%). The median PRL level at diagnosis was 207 (IQR 116.6–1,056.5; range 72–10,000) ng/mL. It was significantly higher in the macroprolactinoma group than in the microprolactinoma group (516 vs. 114.2 ng/mL; *P* < 0.001). The maximum tumor diameter ranged from 4 to 74 mm. It was larger in male patients (21 ± 7 mm; range 7–48 mm) than in female patients (12 ± 3 mm; range 4–74 mm) (*P* = 0.548). Clinical information and MRI findings of patient 12, who had the largest giant prolactinoma (7.4 × 4.6 × 5.6 cm), are presented in the Supplementary Table 1, Supplementary Figure 1.

### Clinical Factors Associated With Macroprolactinoma

The presence of a macroprolactinoma was correlated with male gender (*r* = 0.443, *P* = 0.026), high PRL level at diagnosis (*r* = 0.710, *P* < 0.001), and the presence of panhypopituitarism (*r* = 0.623, *P* = 0.001), but not with age at diagnosis, BMI SDS, height SDS, and the Ki-67 index (Table 2). The median duration of DA therapy was longer in the macroprolactinoma group than in the microprolactinoma group, but the difference was not statistically significant (BRC 0.9 vs. 2.1 years, *P* = 0.333; CAB 0.3 vs. 1.8 years, *P* = 0.081).

**Table 2:** Correlation analysis of clinical parameters for macroprolactinoma.

	***r***	P^*^
Gender (male)	0.443	0.026
Age at diagnosis	−0.073	0.730
Height (SDS)	−0.145	0.488
BMI (SDS)	0.168	0.423
Ki-67 index	0.174	0.536
Nadir PRL level	0.184	0.378
PRL at diagnosis	0.710	<0.001
Panhypopituitarism	0.623	0.001

*r, Spearman rank order correlation coefficient; P, P-value*.

* *Significant association was classified as P < 0.05*.

*BMI, body mass index; SDS, standard deviation score; PRL, prolactin*.

### Responsiveness to Dopamine Agonists

The median follow-up period after diagnosis was 3.0 (range 2.0–4.1) years. Eighteen patients received medical treatment with DAs, 10 of whom were responsive to DAs (56%). Responsiveness to DAs was better in the microprolactinoma group than in the macroprolactinoma group; however, the difference was not statistically significant (71 vs. 45%, *P* = 0.436). Of the eight patients who were resistant or intolerant to DAs, six had macroprolactinomas; of these, three eventually underwent GKS. All patient characteristics and detailed descriptions have been summarized in Supplementary Table 1.

### Surgery vs. Non-surgery Group

Among the 16 patients who underwent TSA, 13 showed macroprolactinomas (13/16, 81%) and the other three showed borderline-sized prolactinomas with a maximum diameter of 10 mm. The reasons for surgery varied, and 24% of patients (*n* = 6, patients 3, 4, 8, 17, 21, and 22) underwent first-line surgery because they presented to a neurosurgeon first and preferred a surgical approach. Other reasons for surgery were resistance to DA (*n* = 3, patients 12, 15, and 25), cystic prolactinoma (*n* = 2, patients 6 and 7), non-adherence to DA (*n* = 1, patient 5), intolerance to DA (*n* = 1, patient 13), premature withdrawal of DA (*n* = 1, patient 16), rapid visual impairment as a result of pituitary apoplexy (*n* = 1, patient 18), and personal preference (*n* = 1, patient 24). Detailed patient descriptions are provided in the Supplementary Data.

The median age at diagnosis was the same (16.9 years) in the surgery and non-surgery groups. The maximum tumor diameter was significantly greater in the surgery group than in the non-surgery group (23.4 vs. 7.9 mm, *P* = 0.002). The PRL level at diagnosis was higher in the surgery group than in the non-surgery group (382 vs. 119 ng/mL, *P* = 0.013). The surgery group showed lower responses to DAs [4/10 patients (25%) vs. 7/8 patients (88%)] and received higher doses with longer periods of DA treatment as compared with the non-surgery group, but no significant differences were noted. The macroprolactinoma group with a higher PRL level at diagnosis (*P* = 0.039) showed a remarkable decrease in the serum PRL level after the TSA (*P* = 0.014) as compared with the findings in the microprolactinoma group.

### Gamma Knife Surgery

Table 3 describes the characteristics of three patients who underwent GKS.

**Table 3:** Characteristics of patients who received gamma knife surgery.

**Patient**	**BMI (SDS)**	**Presenting symptom**	**Ki-67 index (%)**	**Maximum tumor diameter (mm)**	**PRL at diagnosis (ng/mL)**	**Immediate postoperative PRL level (ng/mL)**	**Pre GKS PRL (ng/mL)**	**Post GKS PRL (ng/mL)**	**Tumor volume (mm^**3**^)**	**Prescription dose (Gy)**
P7	2.1	Gal	4.16	20	185	33.6	81.09	58.9	1,021	15
P13	1.4	Gal	3.00	10	78	2.9	64.9	29.4	231	25
P15	1.32	HA	4.0	25	3,950	278	527.4	437.1	5,900	24

*BMI, body mass index; SDS, standard deviation score; PRL, prolactin; GKS, gamma knife surgery; TSA, transsphenoidal approach; GAL, galactorrhea; HA, headache*.

Patient 7, who presented with galactorrhea, was diagnosed with a cystic macroprolactinoma involving the sellar and suprasellar regions. She underwent TSA due to resistance to high-doses of BRC. She then received GKS due to tumor recurrence during long-term CAB treatment and intolerance to DA (nausea, dizziness), and was finally able to discontinue CAB treatment.

Patient 13 presented with galactorrhea and was diagnosed with a 10 mm borderline-sized prolactinoma; she underwent TSA for severe DA intolerance (serious gastrointestinal symptoms, dizziness) even at low-doses of DA that precluded increasing the dosage. While she was doing well without DA after surgery, a residual mass recurred after 2 years, and she eventually received GKS.

Patient 15 was diagnosed with a macroprolactinoma having a maximum diameter of 25 mm owing to severe headache, and he showed a high serum PRL level (3,950 ng/mL) at diagnosis. Despite treatment with the TSA and high-dose CAB, there was no improvement in headache and the PRL level did not decrease below 500 ng/mL. To detect the presence of macroprolactin for the possibility of false hyperprolactinemia, precipitation with 25% polyethylene glycol (diluted at 1:1) was conducted, and true hyperprolactinemia was verified as there was little difference in the PRL level between pre- and post-PEG precipitation (26). He underwent GKS at age 15 years owing to the gradual growth of a remnant tumor encasing the right cavernous internal carotid artery, resulting in optic nerve deformity and sustained hyperprolactinemia. At diagnosis, he had four anterior pituitary hormone deficiencies (ACTH, GH, TSH, and LH/FSH). Interestingly, he showed reversal of ACTH and TSH deficiency after self-discontinuation of hydrocortisone (5.9 mg/body surface area/day), testosterone, and levothyroxine (100 μg once daily) 2 months after GKS. Currently, he shows normoprolactinemia while taking only CAB 1.5 mg every 2 weeks.

All three patients showed a reduced tumor size (more than 90%) and symptom remission after GKS. During the regular endocrine follow-up in these patients, there was no new onset of hypopituitarism.

### Evaluation of Anterior Pituitary Function

A combined pituitary hormone stimulation test was performed at diagnosis in all patients (Table 1). Overall, 11 patients showed normal findings, 12 patients showed multiple anterior pituitary hormone deficiencies (11 with ≥3 anterior pituitary hormone deficiencies and 1 with 2 anterior pituitary hormone deficiencies), and 2 patients showed only ACTH deficiency. The prevalence of anterior pituitary hormone deficiency was as follows: ACTH, 14/25 (56%); GH, 12/25 (48%); TSH, 9/25 (36%); and LH/FSH, 4/23 (17%; 4 patients with hypogonadotropic hypogonadism).

In the macroprolactinoma group, 10 of 14 patients had panhypopituitarism (71%, *P* = 0.008) at diagnosis. The average height of 12 patients with GH deficiency at diagnosis was −0.54 SDS, and the growth rate was improved while undergoing treatment for the prolactinoma [median height at the end of follow-up: −0.1 (IQR −1.9 to +0.7) SDS].

## Discussion

In this study, macroprolactinoma (56% of patients) was more common than microprolactinoma, in contrast to the predominance of microprolactinoma in adults (6, 27), which is consistent with previous literature (10, 11, 28–30). The median age at diagnosis was 16.9 years, which is similar to that in prior studies (10, 28, 30, 31). The median age at diagnosis was lower in male patients than in female patients, and this might be associated with the mass effects of male patients (7, 10, 11, 29, 30, 32, 33).

The degree of hyperprolactinemia is generally known to be associated with the tumor size (34). Macroprolactinoma is usually related to serum PRL levels >250 ng/mL, and when serum PRL levels are higher than 500 ng/mL, macroprolactinoma is almost always diagnosed (35). In our study, which includes only pure PRL-secreting adenoma, the average PRL level was significantly higher in the macroprolactinoma group than in the microprolactinoma group (516 vs. 114 ng/mL).

Hyperprolactinemia might also be related to weight gain which promotes obesity (36, 37). In our study, one patient was obese with BMI >2 SDS. Compared to the 23% weight gain at diagnosis as one reason for seeking medical advice in Salenave et al. (30), obesity was not a key clinical finding in diagnosis for prolactinoma in this study. This difference may be associated with the degree of hyperprolactinemia, gender, age, and racial/ethnic differences.

The long-term prognosis for prolactinoma is not yet clearly defined, but it is known that only 5–10% of microprolactinomas slowly enlarge over a decade (27). Our study also found that among all patients with microprolactinomas, the tumors did not increase in size during the tracking period. On the other hand, macroprolactinomas are known to be associated with poor prognosis. The overall recurrence rate after CAB treatment for a prolactinoma is ~50%, which is known to be associated with tumor size and PRL level at diagnosis (38). The large proportion of macroprolactinomas and great growth potential due to increased proliferative capacity in children and adolescents when compared with the findings for adults also make remission difficult (9). Although DA resistance is more common in macroprolactinomas, the overall reported frequency of resistance to DA is about 25% in pediatric prolactinomas (10, 30, 39). This suggests that the use of a sufficient period of time and maximal dose of DA can obtain successful disease remission among children and adolescents with prolactinomas.

DA withdrawal should also be considered very carefully, however, tapering standards in children are poorly defined. Hoffman et al. (8) reported only one case of successful discontinuation of DA in a child. In our study, successful DA withdrawal was observed in four patients (patients 2, 3, 10, and 11), of which the minimum duration of treatment was 1.2 years. Patient 16 discontinued DA after 5 months of treatment but showed an increase in serum PRL level and tumor size after 3 months, and received TSA due to premature withdrawal failure, and personal preference. Despite the remaining controversy, given that the guidelines suggesting withdrawal of DA after 2 years of treatment in adults (40), withdrawal should also be attempted after long-term treatment in children and adolescents.

Primary treatment for prolactinoma has recently changed from BRC to CAB, which has fewer side effects (such as GI issues and orthostatic hypotension) and greater therapeutic effect. However, risk of cardiac valve disease should be considered and regular echocardiography is required for patients who are taking a weekly dose of more than 2 mg CAB or high cumulative dose (41, 42). Five of our patients (patients 7, 14, 15, 18, and 19) who recently performed echocardiography had no valvular abnormalities.

In this study, the overall response rate to DAs was 61%. The response rates to DAs for microprolactinomas and macroprolactinomas were 71 and 45%, respectively. These values are similar to rates reported by Colao et al. (43) (66.1% for microprolactinomas and 46.9% for macroprolactinomas) and lower than rates reported by Salvenave et al. (30) (74% for macroprolactinoma). This difference could have resulted from differences in the definition of responsiveness to DA and lower maximum doses of DA in our patients compared to other pediatric studies (30, 44). Meanwhile, a high Ki-67 index, which is often recognized as a threshold for presumed invasive prolactinoma and greater recurrence, was not significantly associated with macroprolactinomas or the response to treatment in our cohort (32, 45, 46).

Prolactinomas are known to have a higher rate of post-surgical recurrence within 5 years as compared with other pituitary adenomas (47). The recurrence rate of prolactinomas after the TSA in this study was 31% (5/16), and all of these patients had macroprolactinomas, except one patient who had a prolactinoma with a diameter of 10 mm (Supplementary Table 1). This is higher than the recurrence rate of 13–20% after surgery in adults with prolactinomas (48, 49). This is in line with previous literature mentioning that pediatric patients with secretary pituitary adenomas show more difficulty in achieving remission and are more prone to recurrence as compared with adults (49–52). In our cohort, four patients showed complications associated with the TSA (53), including CSF leakage, meningitis, and transient central diabetes insipidus, which were well-controlled without progression to serious conditions.

The pediatric pituitary adenoma is especially susceptible to hypopituitarism because of the high incidence of recurrence (52), but detection of multiple pituitary hormone deficiencies in prolactinomas has been described in only a few studies (30, 39). In this study, the rate of deficiency was the highest for ACTH (56%), followed by GH (48%), TSH (36%), and LH/FSH (17%) at the time of diagnosis. Four patients showed pubertal delay with hypogonadotropic hypogonadism at diagnosis. Of 12 patients who were diagnosed with GH deficiency, definite short stature (< -2 SDS) was observed in only two patients (patients 6 and 12). In particular, most patients with panhypopituitarism (10/11, 90.9%) had macroprolactinomas, reflecting the mass impact on the pituitary gland (Tables 1, 2) (29, 54, 55). Previous studies involving adult patients with macroprolactinomas identified LH and FSH deficiency in 73–93% of patients, TSH deficiency in 41% of patients, and ACTH deficiency in 12–23% of patients at diagnosis (54, 56), whereas a recent study by Breil et al. (39) involving 12 pediatric patients with prolactinomas identified GH deficiency in 41.7% of patients, TSH deficiency in 33.3% of patients, LH and FSH deficiency in 25% of patients, and ACTH deficiency in 17% of patients. When compared with the pediatric prolactinoma findings of Salvenave et al. (30) (8/77, 10%) and Breil et al. (39) (2/12, 16.7%), the prevalence of panhypopituitarism at diagnosis in this study was higher (11/25, 44%), despite the similar age at diagnosis and proportion of macroprolactinoma. These differences are constrained for interpretation owing to the different characteristics of each study cohort and the scarcity of other comparable data.

GKS is a sophisticated technique performed with a single dose. It allows faster control of hypersecretion and increased focus compared to conventional radiotherapy, particularly when delivering maximal doses to the pituitary and infundibulum (16, 17, 57–59). However, in 20–40% of GKS cases, hypopituitarism is a major adverse effect (60). GKS is mainly used as a secondary therapeutic option after surgery for residual or recurrent pituitary tumors. Although literature on the effect of radiotherapy including GKS for pediatric prolactinoma is scarce (20, 30), the outcome reported in the study by Salenave et al. (30), which showed PRL normalization in three out of four pediatric prolactinoma patients who received radiotherapy, are impressive. In this study, three patients underwent GKS and had improved clinical outcomes without serious complications or newly developed pituitary deficiency. One patient (patient 15) who underwent GKS showed reversal of anterior hormone deficiency, which supports prior results of the influence of GKS on recovery of hormonal function (61–64). Although outcomes of GKS in our young patients were favorable, accurate evaluation of the efficacy of GKS should be performed with long-term accumulated data.

All young patients with prolactinoma in this study were sporadic cases, and most prolactinomas develop sporadically without any known association to a genetic condition (65). However, lack of comprehensive genetic testing is the limitation of this study, as up to 2.6% of patients with prolactinoma without related MEN1 symptoms may experience mutations in the *MEN1* gene (66). This study has other limitations, including a relatively small number of patients and limited information about long-term clinical courses. However, research on pediatric prolactinoma is limited due to its rarity, and there is insufficient existing data. This is a single-center study, so the definition of responsiveness to DA, surgical approach, method for interpreting sella MRI findings, and follow-up protocol are consistent, making the data highly relevant.

## Conclusion

A macroprolactinoma is more prevalent than a microprolactinoma in the pediatric population, contrary to the finding in the adult population. Male gender, increased PRL levels, and the presence of panhypopituitarism at diagnosis are closely related to macroprolactinomas in children and adolescents. Further studies on long-term therapeutic outcomes and a prognostic model are needed to assess prolactinomas in children and adolescents.

## Data Availability Statement

All datasets presented in this study are included in the article/Supplementary Material.

## Ethics Statement

Written informed consents were obtained from patients and their parents of each patient, and the Institutional Review Board at Samsung Medical Center approved the study (IRB file number: 358 2018-06-050).

## Author Contributions

AY designed and supervised the study, overseeing the data collection, interpretation, management, statistical analysis, and drafting of the article for this study. SC contributed to the research design, data analysis and interpretation, the drafting and critical review of the paper, and the approval of the submitted paper. HP developed the structure and arguments for the paper and was also responsible for the collection of clinical data of pediatric prolactinoma patients in our center. MK analyzed and interpreted the data. D-SK and H-JS contributed to the writing of the manuscript as an expert surgeon who performed surgery on our patients. D-KJ was the team leader, secured funding for this project, and also contributed the approval of the submitted paper. SC and D-KJ contributed equally to this work. All authors read and approved the final manuscript.

## Conflict of Interest

The authors declare that the research was conducted in the absence of any commercial or financial relationships that could be construed as a potential conflict of interest.

## Supplementary Material

Supplementary Figure 1 Gadolinium-enhanced T1-weighted magnetic resonance imaging (MRI) images of patient 12 with giant prolactinoma. **(A)** Sagittal view, **(B)** coronal image, **(C,D)** axial images: MRI shows 7.4 × 4.6 ×5.6 cm pituitary tumor invading optic chiasm, bilateral cavernous sinus, and suprasellar area.

Supplementary Table 1 Characteristics of 25 children and adolescents with prolactinoma.

## Abbreviations

PRL: prolactin
GnRH: gonadotropin-releasing hormone
FSH: follicle-stimulating hormone
LH: luteinizing hormone
DAs: dopamine agonists
TSA: transsphenoidal approach
GKS: gamma knife surgery
MRI: magnetic resonance imaging
BMI: body mass index
SDS: standard deviation score
T3: triiodothyronine
free T4: free thyroxine
TSH: thyroid-stimulating hormone
IGF-1: insulin-like growth factor-1
ACTH: adrenocorticotropic hormone
GH: growth hormone
BRC: bromocriptine
CAB: cabergoline
SD: standard deviation
IQR: interquartile range
GI: gastrointestinal.
