# Supplementary figures and images for: Clinical, Hormonal, and Neuroradiological Characteristics and Therapeutic Outcomes of Prolactinomas in Children and Adolescents at a Single Center
Source: Front Endocrinol (Lausanne). 2020 Aug 4;11:527. doi: 10.3389/fendo.2020.00527 (PMC7417303; doi:10.3389/fendo.2020.00527)

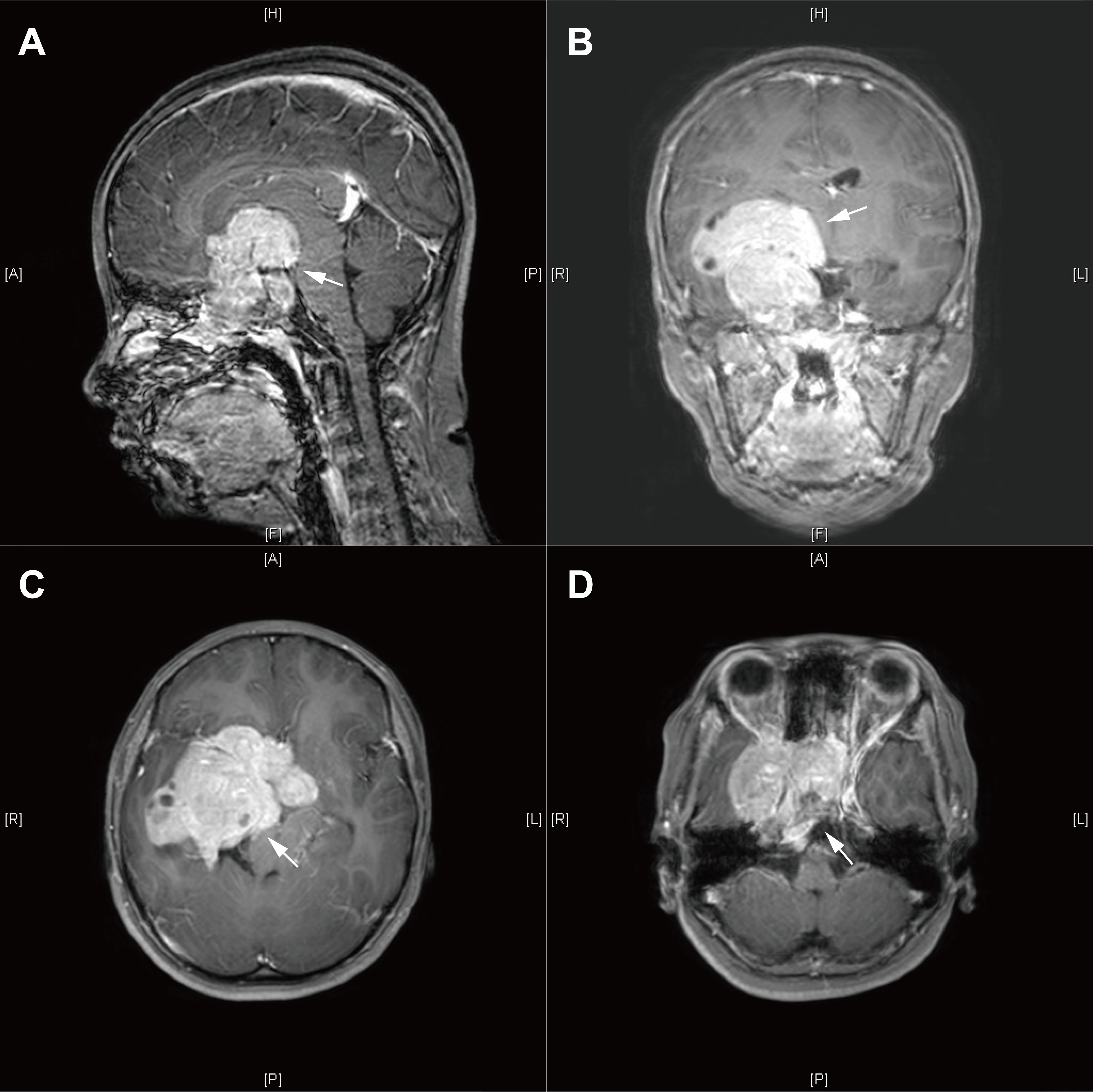

Supplement: Supplementary Figure 1 — Gadolinium-enhanced T1-weighted magnetic resonance imaging (MRI) images of patient 12 with giant prolactinoma. (A) Sagittal view, (B) coronal image, (C,D) axial images: MRI shows 7.4 × 4.6 ×5.6 cm pituitary tumor invading optic chiasm, bilateral cavernous sinus, and suprasellar area. [file Image_1.JPEG]
