# Supplementary material for: Clinical, Hormonal, and Neuroradiological Characteristics and Therapeutic Outcomes of Prolactinomas in Children and Adolescents at a Single Center
Source: Front Endocrinol (Lausanne). 2020 Aug 4;11:527. doi: 10.3389/fendo.2020.00527 (PMC7417303; doi:10.3389/fendo.2020.00527)
Supplement: Supplementary Table 1 — Characteristics of 25 children and adolescents with prolactinoma. [file Table_1.docx]

**Supplementary Table |** Characteristics of 25 children and adolescents with prolactinoma.

| **Patient** | **Symptom at diagnosis** | **Height SDS** | **BMI SDS** | **Anterior pituitary hormone deficiencies** | **PRL at diagnosis (ng/mL)** | **Nadir PRL**  **(ng/mL)** | **Postop decline of PRL level (%)** | **Ki-67 index (%)** | **Maximum tumor diameter (mm)** | **Maximal dose*,**  **CAB/BRC** | **Responsiveness to DAs** | **Tumor shrinkage (%)** | **Follow-up (yr)** | **Treatment course** |
| --- | --- | --- | --- | --- | --- | --- | --- | --- | --- | --- | --- | --- | --- | --- |
| 1 | GAL | 0.56 | -0.88 | n | 72 | 0.2 | N/A | N/A | 5 | N/A | n/a | 90 (spontaneously) | 1.8 | close observation |
| 2 | GAL | 2.04 | 0.36 | N | 189.5 | 63.7 | N/A | N/A | 9 | 1 / 7.5 | y | 67 | 3.0 | BRC (9mo)🡪 CAB (8mo)🡪 d/c |
| 3 | VFD | -0.41 | 1.36 | Acth, GH, TSH | 207 | 7.87 | 85.6 | 3.1 | 25 | 0.5 / 3.75 | y | 60 | 5.7 | TSA(17yr)🡪BRC (2.2yr)🡪cab(3.1yr)🡪D/C |
| 4 | AMN | -1.80 | -0.88 | Acth, GH, TSH | 230 | 9.5 | 98.8 | 0.1 | 10 | n/a | n/a | 100 | 3.2 | tsa(16.5yr) |
| 5 | HA | 0.98 | 0.41 | Acth, GH, TSH, | 3440 | 10 | 97.6 | 2.2 | 20 | / 10 | y | 100 | 10.4 | brC (5mo)🡪tsa(15yr)🡪BRC (8.1yr~) |
| 6 | VFD | -2.80 | -0.27 | Acth, gh, LH/FSH | 7146 | 88 | 85.2 | 1.6 | 21 | / 210 | r | 30 | 3.6 | BRC(10mo)🡪 tsa (19yr)🡪brC(1.6yr) |
| 7 | GAL | 0.25 | 2.11 | Acth, gh, LH/FSH | 185 | 36 | 81.9 | 4.2 | 20 | 2 / 52.5 | R | recur | 8.4 | brC(1yr)🡪 tsa(19.5yr)🡪 cAB (5.7yr)🡪 GKS |
| 8 | AMN | 0.65 | -0.45 | Acth, GH | 452 | 2.3 | 99.7 | 1.2 | 12 | N/A | N/A | 100 | 2.5 | TSA (18.1yr) |
| 9 | GAL | -1.65 | 0.26 | n | 119 | 0.1 | N/A | N/A | 4 | 2 / | r | n/a | 3 | CAB (3.2yr) |
| 10 | AMN | -0.55 | 0.22 | n | 105 | 33 | N/A | N/A | 7 | 1 / | Y | 71 | 2 | CAB (1.2yr)🡪D/C |
| 11 | GAL | 1.69 | 0.44 | n | 112 | 0.56 | N/A | N/A | 9 | 0.25 / 2 | Y | 56 | 3 | BRC (2mo)🡪CAB (1.5yr)🡪D/C |
| 12 | HA | -2.67 | -0.68 | Acth, GH, TSH | 10000 | 65 | 90.0 | 13 | 74 | 2 / 70 | r | recur | 5.3 | BRC (2.1yr)🡪TSA(16.5yr)🡪CAB (1.7yr) |
| 13 | GAL | 0.41. | 1.40 | Acth | 78 | 2.9 | 96.3 | 3 | 10 | 1 / 2.5 | I | recur | 5.3 | brC(6mo) 🡪 cAB (6mo)🡪 tsa(19.7yr)🡪gks |
| 14 | AMN | -1.45 | -0.17 | n | 230 | 0.1 | N/A | N/A | 12 | 2 / 2.11 | y | 75 | 4.2 | brC(1wk)🡪D/C cAB(3.2yr) |
| 15 | HA | -0.88 | 1.32 | Acth, GH, TSH, LH/FSH | 3950 | 278 | 93.0 | 4 | 25 | 4 / | r | recur | 3.8 | cAB(2.8yr)🡪tsa (13.4yr)🡪gks |
| 16 | AMN | -0.14 | -0.14 | n | 312 | 9.4 | 93.8 | 6.3 | 15 | / 1.0 | Y | 100 | 3.9 | brC (5mo)🡪 D/C  Tsa (18.2yr) |
| 17 | AMN | 0.48 | 0.66 | aCTH | 190 | 5.8 | 97.4 | 3.7 | 12 | n/a | n/a | 100 | 2.8 | tsa (16.6yr) |
| 18 | VFD | -1.92 | 0.07 | Acth, GH, TSH | 1533 | 8.5 | 95.0 | 16 | 30 | 1.5 / | r | 35 | 2.6 | tsa (17.7yr)🡪 cAB (1.4yr) |
| 19 | HA, AMN | -0.45 | 0.02 | N | 406 | 13.4 | N/A | N/A | 9 | 2 / | Y | 67 | 2.0 | cAB (1.1yr) |
| 20 | GAL | 0.31 | 0.61 | N | 114.2 | 45 | N/A | N/A | 9 | 1.5 / | n/a | N/A | 2.0 | cAB (1mo)🡪D/C |
| 21 | GAL | -0.11 | 0.39 | N | 85.6 | 3.4 | 98.5 | 5 | 10 | N/A | N/A | 100 | 2.0 | TSA (14yr) |
| 22 | AMN | 0.70 | 1.00 | Acth, GH, TSH, LH/FSH | 162 | 10.4 | 94.4 | 3 | 22 | n/a | n/a | 100 | 2.1 | tsa (18.6yr) |
| 23 | GAL | 2.19 | 0.15 | N | 172.5 | 7.5 | N/A | N/A | 7 | 1 / | Y | N/A | 1.6 | cAB (7mo) |
| 24 | GAL, AMN | 0.70 | 1.75 | Acth, GH, TSH | 580 | 0.6 | 95.7 | 2 | 21 | 1 / | Y | 70 | 1.7 | CAB (6mo)🡪 TSA (16.7yr) |
| 25 | VFD | 0.70 | 0.56 | Acth, GH, TSH | 3013 | 18.4 | N/A | 2.8 | 51 | 2.5 / | R | recur | 2.0 | CAB (1YR)-> tsa (12yr) |

*GAL, galactorrhea; AMN, amenorrhea; VFD, visual field defect; HA, headache; CAB, cabergoline; BRC, bromocriptine; ACTH, adrenocorticotropic hormone; GH, Growth hormone; TSH, thyroid-stimulating hormone; LH, luteinizing hormone; FSH, follicle-stimulating hormone; SDS, standard deviation score; PRL, prolactin; TSA, transsphenoidal approach; DAs, dopamine agonists; yr, years; mo, months; D/C, discontinue; N/A, not applicable; Y, responsive; R, resistant; I, intolerant*

**unit: CAB, mg/week and BRC, mg/day.*

**Detailed patient information**

**Patient 3** first visited the department of neurosurgery and underwent surgery. Afterward, normalization of serum PRL level was maintained with median 3.75 mg/day of BRC. Since then, BRC changed to CAB according to recent medical trends, and PRL levels were well maintained for three years and one month even at small doses of CAB, which was eventually discontinued.

**Patients 4, 8, 17, 21, and 22** were first referred to a neurosurgeon and underwent surgery in combination with patient preference. These patients showed only positive for PRL in immunohistochemistry. Symptoms improved and normalized PRL levels remained for over six months, which did not warrant additional DA therapy.

**Patient 12** presented with headache and was diagnosed with giant prolactinoma (7.4 x 4.6 x 5.6 cm) involving optic chiasm, bilateral cavernous sinus, and suprasellar area (Figure S1). Ki-67 index was 13%, and initial PRL level at diagnosis was above 10000 ng/mL. This patient received TSA due to resistance to BRC (up to 70 mg/day) and tumor recurrence.

**Patient 16** presented with amenorrhea and had favorable response to low-dose BRC. Early drug discontinuation was attempted, but the patient underwent TSA due to personal preference and increased serum PRL level and tumor size following premature withdrawal.

**Patient 18** was referred to a neurosurgeon first and was diagnosed with macroprolactinoma (maximal diameter: 30 mm) bulging into the cavernous sinus and displacing the left internal carotid artery by mass effect. The serum PRL level at diagnosis was as high as 1,533 ng/mL, and the Ki-67 index was 16%. Since the patient was suspected to have rapid visual impairment resulting from pituitary apoplexy, he received TSA prior to DA. He has been treated with CAB since receiving TSA one month after diagnosis, but his visual impairment was not improved, there was no sustained normalization of PRL, and nausea from CAB continued.

**Patient 24** showed responsiveness to DA with >70% reduction in tumor volume and sustained PRL normalization over six months, even at 1 mg/week of CAB. However, neither the patient nor his parents wanted to continue DA treatment for personal reasons, and strongly preferred surgery.

**Patient 25,** a male patient presenting with visual field defect, was diagnosed with giant prolactinoma (tumor volume: 5.1 x 3.1 x 3.2 cm). At presentation, the mass involved the sella and suprasella areas, extended downward to the sphenoid sinus, bulged into the bilateral cavernous sinus, caused luminal narrowing of the right internal carotid artery, and extended upward to the third ventricle. The patient underwent TSA because there was no noticeable improvement despite one year of 2.5 mg CAB treatment. After TSA, tumor volume reduced by >85% and PRL had sustained normalization; therefore, CAB was eventually discontinued. Nonetheless, the patient experienced severe bilateral optic nerve atrophy damage from early mass effect and was eventually nearly blind.
